# Exploring the variation in implementation of a COPD disease management programme and its impact on health outcomes: a *post hoc* analysis of the RECODE cluster randomised trial

**DOI:** 10.1038/npjpcrm.2015.71

**Published:** 2015-12-17

**Authors:** Melinde R S Boland, Annemarije L Kruis, Simone A Huygens, Apostolos Tsiachristas, Willem J J Assendelft, Jacobijn Gussekloo, Coert M G Blom, Niels H Chavannes, Maureen P M H Rutten-van Mölken

**Affiliations:** 1 Institute for Medical Technology Assessment, Erasmus University, Rotterdam, The Netherlands; 2 Institute of Health Policy and Management, Erasmus University, Rotterdam, The Netherlands; 3 Department of Public Health and Primary Care, Leiden University Medical Center, Leiden, The Netherlands; 4 Health Economics Research Centre, Department of Population Health, University of Oxford, Oxford, UK; 5 Department of Primary and Community Care, Radboud University Nijmegen Medical Center, Nijmegen, The Netherlands; 6 Zorgdraad Foundation, Wijnand van Arnhemweg 54, Oosterbeek, The Netherlands

## Abstract

This study aims to (1) examine the variation in implementation of a 2-year chronic obstructive pulmonary disease (COPD) management programme called RECODE, (2) analyse the facilitators and barriers to implementation and (3) investigate the influence of this variation on health outcomes. Implementation variation among the 20 primary-care teams was measured directly using a self-developed scale and indirectly through the level of care integration as measured with the Patient Assessment of Chronic Illness Care (PACIC) and the Assessment of Chronic Illness Care (ACIC). Interviews were held to obtain detailed information regarding the facilitators and barriers to implementation. Multilevel models were used to investigate the association between variation in implementation and change in outcomes. The teams implemented, on average, eight of the 19 interventions, and the specific package of interventions varied widely. Important barriers and facilitators of implementation were (in)sufficient motivation of healthcare provider and patient, the high starting level of COPD care, the small size of the COPD population per team, the mild COPD population, practicalities of the information and communication technology (ICT) system, and hurdles in reimbursement. Level of implementation as measured with our own scale and the ACIC was not associated with health outcomes. A higher level of implementation measured with the PACIC was positively associated with improved self-management capabilities, but this association was not found for other outcomes. There was a wide variety in the implementation of RECODE, associated with barriers at individual, social, organisational and societal level. There was little association between extent of implementation and health outcomes.

## Introduction

Integrated Disease Management (DM) is a popular approach for improving the quality and efficiency of care in chronic obstructive pulmonary disease (COPD) patients. However, the key elements of DM programmes for COPD (herein, COPD-DM) are not yet fully understood.^1–3^ The cost-effectiveness of these programmes varies considerably,^4^ most likely depending on the duration, target population and components of the intervention.^5,6^ Moreover, wide variation exists, even in the implementation of a single programme.^7,8^ This variation can be due to adjustments for the local setting, or due to differences in specific barriers and facilitators that influence implementation.^9,10^ Therefore, it is important to understand the conditions needed for the successful implementation of a DM programme.^11^

We aimed to (i) examine the variation in implementation of a single COPD-DM programme (RECODE) between different primary-care teams, (ii) analyse the facilitators of and barriers to implementation and (iii) investigate the association between the extent of implementation and health outcomes. This study was performed as a pre-specified part of the RECODE trial.^12^

## Results

Table 1 summarises the characteristics of the teams and their COPD patients. Each team enrolled 11–55 patients and 53 percent of the teams were delivering *ad hoc* reactive care.

The telephone interviews were held with five general practitioners (GPs) and 17 practice nurses from 17 of the 20 (85%) teams. These interviews varied in length between 20 and 45 min. Three (15%) teams could not be interviewed because the participating caregiver(s) had left or changed practice or because the caregiver(s) lacked time. The response rate of the questionnaires can be found in Appendix 2.

### Implementation

The teams implemented, on average, 8 of the 19 interventions (range: 2–14, Table 2). The most frequently applied interventions were cooperation with physiotherapist(s) (88%), exacerbation management (76%) and active identification and monitoring of high-risk COPD patients (71%). Only a few teams improved cooperation with lung specialist(s) (18%), substituted care from secondary to primary care (24%), actively applied motivational interviewing to improve self-management (18%) and used additional funding for physiotherapy (12%). In the second study year, none of the teams used the ITC system 'Zorgdraad'. Teams with a lower starting level implemented, on average, more interventions than did teams with a higher starting level.

The total Patient Assessment of Chronic Illness Care (PACIC) score did not significantly change over the study period (Table 3). However, the PACIC component ‘decision support’ significantly decreased. Even though the total Assessment of Chronic Illness Care (ACIC) score did not significantly change, the ACIC components ‘organisation of healthcare system’, ‘community linkages’ and ‘self-management’ significantly improved over the first year.

### Barriers and facilitators

Table 4 summarises the barriers and facilitators to implementation as they were perceived by the teams grouped into individual, social, organisational and broader societal factors. These groups were not mutually exclusive.

### Individual factors

Caregivers were positive about the RECODE course, stating that it was informative, increased attention to COPD, and inspired and motivated them to improve COPD care. For instance, 13 teams reported a greater awareness of early recognition of exacerbations, and many teams implemented symptom-reporting policies to increase early treatment of exacerbations. Teams also fine-tuned self-management, which was already (partly) integrated. For instance, most teams were familiar with motivational interviewing and individual treatment plans, but not every plan was put in writing or was made in consultation with the patient.

A barrier to implementation was the lack of motivation of patients. Several teams reported difficulties in persuading COPD patients to adopt healthier behaviour because they did not feel ill or did not experience (many) problems. Furthermore, because of the lack of patients’ motivation, familiarity with computers and obligation to use web-based applications, few patients used Zorgdraad. The lack of motivation or time to determine how Zorgdraad worked was also an important barrier among caregivers in using Zorgdraad.

### Social factors

During the refresher courses, the teams discussed their implementation experiences with other teams. That way they motivated, inspired and learnt from each other. These presentations were generally appreciated, although some were dissatisfied that presenters had begun implementation rather late, as a result of which they had little experience to share.

The professional network in which the teams operated was also a barrier to implementation. For instance, inconsistent use of Zorgdraad among team members jeopardised the potential contribution of Zorgdraad to their purposes.

### Organisational factors

Teams with a lower starting level had more room for improvements. For example, only teams with no structured COPD care developed new protocols. Furthermore, four teams changed smoking cessation support because most teams reported that this was already integrated in their COPD care. Moreover, four teams did not re-allocate tasks from the GP to the practice nurse because they reported that most of the COPD care had already been re-allocated to the practice nurse.

The implementation was also facilitated by the feedback reports on the health outcomes of their patients that each practice received. Several teams indicated a better overview and greater ability to manage progression of their COPD patients. In this way, the reports helped to actively track high-risk COPD patients.

Four teams reported changes in referring patients to primary/secondary care. Three teams explicitly discussed referral criteria, whereas in one team the lung specialist noticed changes in primary care and referred more patients back without explicit deliberation. A barrier for task re-allocation from secondary to primary care was that not every lung specialist adhered to the new agreements.

The main barrier to improving cooperation with the dietitian was the low proportion of patients who were eligible to be referred for nutritional support. The low number of patients and staff turnover was also reported as reasons for not using Zorgdraad and organising periodically scheduled multidisciplinary meetings. In addition, problems with transferring information to the team’s information system was an important barrier in Zorgdraad.

### Broader societal factors

A bundled payment scheme for COPD patients was introduced in the Netherlands, almost simultaneously with the start of RECODE.^13^ Since this reform, health insurers purchase integrated multidisciplinary COPD care from care groups. As a result, the focus on COPD care increased, financial coverage improved, and/or secondary caregivers became more involved. This facilitated the implementation of RECODE. However, three teams reported that they abandoned or temporarily stopped RECODE because they had to concentrate on preparing the integrated care programme as was purchased by the insurer and the formal installation of a care group. A care group is a legal entity, usually owned by GPs, which subcontracts individual professionals to provide care.

The lack of full reimbursement of physiotherapy and smoking cessation support was an important barrier to patient participation in these RECODE components. The lack of reimbursement of physiotherapy was partly solved by the RECODE research team, which arranged supplementary funding by healthcare insurers for COPD-specific exercise training programmes for patients with an MRC (Medical Research Council) dyspnoea score >2, including those without supplementary health insurance. The reason for the limited use (12%) of the funding remains unclear. One respondent stated that the funding was not used because attention to RECODE declined, and many patients did not qualify for the reimbursement.

### Association between level of implementation and health outcomes

Table 5 shows the association between the level of implementation of RECODE (as measured either by our own implementation scale, by the change in PACIC or the change in ACIC) and the change in health outcomes within the same time period. A higher level of integrated care as measured by the self-developed scale was not associated with better health outcomes (Table 5). The indirect assessment of implementation, measured as the change in the level of integrated care from the patient’s perspective (PACIC), was associated with a significantly higher ‘SMAS (Self-Management Ability Scale), taking initiatives’ score and a significantly higher ‘SMAS, investment behaviour’ score. For example, one unit improvement in PACIC score between baseline and 24 months was associated with a 1.2 unit improvement in ‘SMAS taking initiative score’ between baseline and 24 months. This association was not found in other health outcomes. Over the 1-year study period, the total score on changed level of integrated care from the healthcare provider’s perspective (ACIC) was not associated with better health outcomes. Within the subgroup of patients with a clinically relevant improvement on the CCQ (Clinical COPD Questionnaire) or SGRQ (St George Respiratory Questionnaire), a higher level of integrated care (self-developed scale, PACIC or ACIC) was not associated with better health outcomes.

## Discussion

### Main findings

This study showed that a pragmatic (non-experimental) implementation of a COPD-DM programme resulted in a low level and a wide variety of implementation across different teams. Important barriers to implementation were insufficient motivation of patients, high starting level of COPD care, small size of the COPD population per team, mild COPD population, practicalities of the ICT system, and hurdles in the reimbursement. Level of implementation as measured with our own scale and the ACIC was not associated with health outcomes. A higher level of implementation measured with the PACIC was positively associated with improved self-management capabilities, but this association was not found for other outcomes.

### Interpretation of findings in relation to previously published work

In line with previous pragmatic studies,^14,15^ the teams implemented various interventions, but none implemented all interventions. Indeed, on average, less than half (42%) of the interventions were implemented despite the fact that the individual interventions have been shown to improve health outcomes.^1,5,16,17^ These findings further support the idea of Pinnock *et al*^18^ who suggest that after proven efficacy the translation of interventions into a practical service should be evaluated in an implementation study. This translation seems to result in lower but more realistic outcomes of the interventions.^19,20^

RECODE was facilitated by informed and motivated caregivers, which corroborates an earlier study.^10^ Despite this, the caregivers were not able to implement all the interventions. In addition, they became demotivated because Zorgdraad was not adequately functioning on time.

COPD patients were not always motivated to change; as COPD patients perceive their suboptimal health status as ‘normal’, COPD had become a way of life.^21^ Excluding unmotivated patients may improve the (cost-)effectiveness of COPD-DM programmes. Specific interventions to change the motivational status of patients are therefore required.

A barrier for implementation was the low potential for improvement owing to the high starting level of COPD care and the mild COPD population. The absence of improvements owing to already high levels of COPD care was pointed out in earlier primary-care trials.^22,23^

The perceived usefulness of Zorgdraad was low. The teams that did use Zorgdraad experienced problems with practicalities and variability in adoption of the system between team members. Furthermore, teams reported unclear instructions and a lack of time or motivation to determine how Zorgdraad worked. A large review corroborates that usefulness, compatibility with work and time were important barriers for the implementation of an ICT system.^24^

The last important barrier to implementation was the hurdle in reimbursement. As teams reported the formation of care groups as facilitators, the ongoing wide implementation of the bundled payment system in the Netherlands might be a positive step towards solving the reimbursement issue. In this system, healthcare insurers purchase integrated multidisciplinary COPD care from care groups.^25^ However, in practice, the financed package varies widely, and therefore not all multidisciplinary care required by a COPD patient is included.^26^

In accordance with our results, previous studies have demonstrated that an improved PACIC score improved self-management.^14,27^ Despite this, our self-developed scale or ACIC score was not associated with better health outcomes. Therefore, implementation of only a few interventions by some teams does not guarantee improvements in patient outcomes in comparison with other teams.

### Strengths and limitations of this study

This study has several strengths. First, a broad range of outcome measures and implementation measurements including different perspectives were used. Second, independent scoring of the self-developed scale ensured the objectivity of the results. Third, the interviewer was not involved in the core research team, which reduced the pressure to give desirable answers.

This study also has several weaknesses. It was not possible to compare the implemented interventions of the intervention teams with the control teams because, to prevent an additional intervention effect, we did not evaluate changes in COPD care in the control group. Therefore, it was not always possible to determine whether changes were caused by RECODE or other factors, such as parallel projects. Second, most interviews were held with only one representative of the team. However, we interviewed practice nurses or GPs, who were the project leaders and provided the best overview of COPD care in their team. Third, the response rate on the ACIC questionnaire at 12 months was low (65%). However, the ACIC score at baseline did not differ much between the responders and the non-responders.

### Implications for future research, policy and practice

This study showed that a pragmatic COPD-DM programme that primarily targets caregivers seems to result in only modest improvements in care. We learnt that focus should be more on patient-oriented interventions. Hence, multiple COPD-DM programmes have shown that patient-oriented interventions or a combination of patient-oriented, provider-oriented and organisational interventions lead to significant improvements in health outcomes.^14,28^ Furthermore, the interventions should be tailored to patients’ needs, skills and preferences, which will imply that, on average, a COPD-DM programme for milder COPD patients will include fewer or less-intensive interventions than a COPD-DM programme for more severe patients.

The room for improvement and the proportion of motivated patients is higher among a selection of COPD patients with a high disease burden. However, focussing on more severe COPD reduces the number of patients who participate in the programme. Consequently, the motivation of professionals to invest time in optimising the programme and negotiating with health insurers on reimbursement of the programme may decrease. It is a challenge for further programmes to find the right balance between sufficient room for improvement and economies of scale. In finding this balance, we should account for the fact that long-term gains can be increased if we can prevent moderate COPD patients from progressing to severe COPD.

### Conclusions

This study adds valuable input to the discussion on development and implementation of COPD-DM programmes. We observed a low level and wide variability of implementation across different primary-care teams. Barriers and facilitators of the implementation were related to factors at individual, social, organisational and broader societal level. There was little association between the level of implementation and improved health outcomes.

## Materials and methods

### Intervention

**Figure 1:**
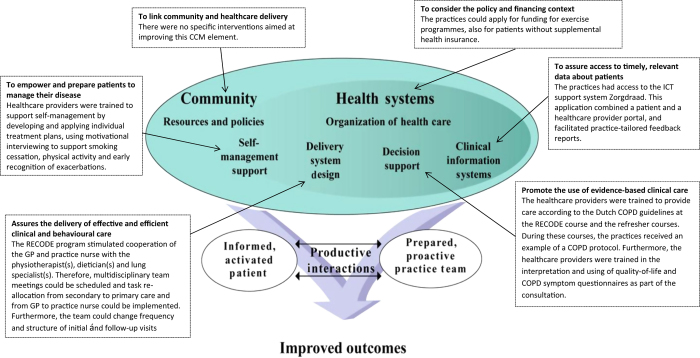
The RECODE interventions grouped by the components of the Chronic Care Model (CCM) from Wagner *et al*.^30^ COPD, chronic obstructive pulmonary disease; GP, general practitioner; ICT, information and communication technology.

RECODE is a two-year cluster-RCT in which 40 primary-care teams were randomised to DM or usual care.^12^ The 20 intervention teams received a 2-day training course in essential elements of effective COPD-DM. These elements are grouped by components of the Chronic Care Model (CCM), and described in Figure 1. The CCM is often used as conceptual framework for development and evaluation of DM programmes.^1,5,16,29^ The core of the CCM is the productive interaction between informed, activated patients and prepared, proactive teams of caregivers.^30^ RECODE included interventions to improve five of the six interrelated CCM components. After the course, the teams were invited to join two refresher courses and had access to the ICT system ‘Zorgdraad’. All the teams were encouraged to write their own reform plan and tailor implementation strategies to their local circumstances. Therefore, the package of interventions that patients received was not only dependent upon their health status, personal needs and preferences but also on local adaptation and level of implementation of interventions.

The ICT system ‘Zorgdraad’ included a patient portal and a healthcare provider portal, but was not an e-consultation system. The patient portal contained educational material and had a section containing personal treatment goals and room to write down personal notes. The provider portal had room for a protocol to guide frequency and content of COPD monitoring, entering quality-of-life scores and results from follow-up and examinations. Information from Zorgdraad was used to generate practice-tailored feedback reports on patients’ health outcomes at baseline and at 6 and 12 months. These reports were generated by the researchers and sent to the practices to support prioritising the healthcare needs. It was intended that practice nurses would give the COPD patients instructions and information through the patient portal.

### Participants

The 20 intervention teams included at least one GP, one practice nurse and one physiotherapist specialised in COPD care. Thirteen teams also included a dietitian. The teams enrolled 554 COPD patients according to the Global Initiative for COPD (GOLD) guidelines,^31^ and because few exclusion criteria were applied, they represent the primary-care COPD population in the Netherlands.^12^

### Setting

In the Netherlands, GPs act as gatekeepers to hospital care; patients need a referral from the GP to visit a specialist in a hospital clinic.^32^ Hence, the vast majority of COPD patients are treated by the GP. The Ministry of Health activity has been stimulating the implementation of integrated care programmes for chronic diseases such as COPD for quite some time. This was reinforced by the introduction of a bundled payment system in 2010.^13^ This has strengthened the collaboration between different primary-care professionals involved in COPD care. Primary-care practice nurses have a key role in providing integrated care. A practice nurse is a new profession that was introduced in the early 2000s, and several tasks formerly performed by GPs were shifted towards this nurse.^32^ The majority (80%) of the practice nurses have a general background in nursing and receive additional training in one or more chronic diseases. They are predominantly involved in the care of chronically ill patients. For COPD patients this includes, for example, periodic monitoring, spirometry testing, inhalation instructions, smoking cessation counselling, coaching patients to become more physically active, and teaching patients to recognise exacerbations early.^33^ At present, 80% of the Dutch practices, which have an average practice size of 2,350 patients, have at least one practice nurse who takes care of chronically ill patients for at least 2 days a week.

### Implementation

The level of implementation was measured directly with a self-developed scale. The scale measured the implementation of 19 interventions in five CCM components that were included in the RECODE programme (Appendix 1). Three researchers independently assessed whether an intervention was actually implemented (score=1) or not (score=0), and disagreements were discussed in a consensus meeting. The sum of these 19 scores comprised the total score on the self-developed scale. The information that the researchers used to score the scale was obtained from a questionnaire administered to the teams after 1 year and a semi-structured telephone interview with the teams after 2 years. Questions were asked about COPD care before RECODE, changes in COPD care as a result of RECODE, and barriers to and facilitators of implementation. The interviews were recorded and transcribed verbatim. Finally, information was recorded on attendance of professionals at the training and refresher courses, as well as on ICT use and use of additional reimbursement for physiotherapy.

The level of implementation was also measured indirectly through the assessment of the level of integrated care that was achieved. The latter was measured from the patient’s perspective with the PACIC Questionnaire^34^ at baseline and at 6, 9, 12, 18 and 24 months (ranging from 1 (lowest level) to 5 (highest level)) and from the healthcare provider’s perspective using the ACIC (ranging from 0 to 11, with 11 representing optimal care) at baseline and at 12 months.^35^

### Barriers and facilitators

Reported barriers and facilitators of implementation were categorised as individual, social, organisational or broader societal factors.^36^ Individual factors were related to caregivers and consisted of cognitive, motivational and behavioural factors, as well as personal characteristics including health status. Social factors were related to professional teams/networks. Organisational factors included the organisational structure, culture and work processes, as well as the availability of necessary resources. Societal factors related to the healthcare system and to societal and political developments.

### Starting level

We distinguished three starting levels: (i) *ad hoc* reactive COPD care; (ii) structural diagnosis of COPD patients; and (iii) structural diagnosis and proactive follow-up of COPD patients. Teams with ‘*ad hoc* reactive COPD care’ had (virtually) no DM. For these teams, RECODE marked the start of structured COPD care. Teams with ‘structural diagnosis of COPD patients’ had begun to structure their COPD care, performed spirometry and had an overview of the COPD population in their practice. Teams with ‘structural diagnosis and proactive follow-up of COPD patients’ additionally had an established control-visit/follow-up structure, and applied strategies to support self-management.

### Health outcomes

We measured health-related quality of life on the CCQ,^37^ SGRQ^38^ and the EQ-5D.^39,40^ We measured dyspnoea by means of the MRC dyspnoea score with a scale from 1 to 5,^41^ physical activity by means of the International Physical Activity Questionnaire^42^ and self-management abilities by means of the components ‘taking initiatives’, ‘investment behaviour’ and ‘level of self-efficacy’ from the Self-Management Ability Scale-30.^43^ The questionnaires were administered at baseline and at 6, 9, 12, 18 and 24 months.

### Statistical analysis

Descriptive statistics of patients’ and teams’ characteristics were calculated. We used two-tailed, paired *t*-tests to investigate improvements in the level of integrated care as measured by the PACIC and the ACIC.

During the RECODE study, there was a variation in changed outcomes in the intervention group.^44^ In this study, we investigated the association between variation in implementation as measured with our own developed scale and ACIC and change in health outcomes within the same time period using two-level (patients nested in teams) linear mixed-effect models, correcting for starting score of different health outcomes and starting level of COPD care. To investigate the impact of the level of implementation as measured with the PACIC on change in health outcomes within the same time period, we used three-level (longitudinal measurements nested in patients nested in primary-care teams) linear mixed-effect models, correcting for time, starting score of different health outcomes and starting level of COPD care. We used six time points (baseline, 6, 9, 12, 18 and 24 months) to estimate the impact of implementation as measured with the PACIC. These models were specified for eight dependent variables: change in CCQ, SGRQ, EQ-5D, MRC, MET minutes, taking initiatives, investment behaviour and self-efficacy.

## Figures and Tables

**Table 1:** Sample characteristics

*Characteristics of the primary-care teams (*N*=20)*	
Practice location, urban, *n* (%)	14 (70)
Practice type, single-handed practice, *n* (%)	8 (40)
Practice type, one or more partner practice, *n* (%)	9 (45)
Practice type, healthcare centre, *n* (%)	3 (15)
Patient practice population, *n* (range)	3,900 (1,900–8,100)
Participating COPD patients, (range)	28 (11–55)
Ethnic minorities, %	16 (1–60)
Years practising GP, y	13 (3–25)
Starting level^a^	
*Ad hoc* reactive COPD care, *n* (%)	9 (53)
Structural diagnosis of COPD patients, *n* (%)	4 (24)
Structural diagnosis and proactive follow-up of COPD patients, *n* (%)	4 (24)

*Patient characteristics (*N*=554)*	
Men, %	50.5
Age (mean, s.d.)	68.2 (11.3)
GOLD stage I, %	25.3
GOLD stage II, %	52.6
GOLD stage III, %	19.0
GOLD stage IV, %	3.1
CCQ (mean, s.d.)	1.54 (0.98)
SGRQ (mean, s.d.)	36.7 (21.1)
EQ-5D (mean, s.d.)	0.74 (0.25)
MRC (mean, s.d.)	2.06 (1.30)
MET minutes (mean, s.d.)	3,101 (4,652)
SMAS, taking initiatives (mean, s.d.)	56.8 (18.1)
SMAS, investment behaviour (mean, s.d.)	61.4 (17.0)
SMAS, self-efficacy (mean, s.d.)	66.0 (17.2)

Abbreviations: CCQ, Clinical COPD Questionnaire; EQ-5D, EuroQoL-5D; MET, metabolic equivalent time; MRC, Medical Research Council; SGRQ, St George’s Respiratory Questionnaire; SMAS, Self-Management Ability Scale.

a Starting level was missing in three teams.

**Table 2:** Implementation of 19 interventions of integrated COPD care over a 2-year follow-up period per primary-care team

*Interventions*	Teams																	*Total*
	I	II	III	IV	V	VI	VII	VIII	IX	X	XI	XII	XIII	XIV	XV	XVI	XVII	
*Delivery system design*																		
Improved cooperation with physiotherapist(s)	0	0	1	1	1	1	1	1	1	1	1	1	1	1	1	1	1	15
Improved cooperation with dietician(s)	0	0	0	0	1	1	0	1	1	1	1	0	1	1	1	1	0	10
Improved cooperation with lung specialist(s)	0	0	0	0	0	0	1	0	0	0	0	0	0	1	0	1	0	3
More multidisciplinary PCT meetings	0	0	1	0	0	0	0	0	0	1	0	0	0	1	0	1	1	5
Task re-allocation from GP to practice nurse or specialised nurse	0	1	0	1	0	0	0	1	0	0	0	1	0	0	1	1	1	7
Substitution of care from secondary to primary care	0	0	0	0	0	0	0	0	0	0	0	0	1	0	1	1	1	4
Change in follow-up and visit structure	0	0	0	0	1	0	0	1	0	0	1	1	1	1	1	1	1	9

*Decision support*																		
Attendance of four disciplines at the initial RECODE course	0	0	1	0	0	0	1	1	0	1	1	1	0	1	0	1	1	9
Attendance of two or more disciplines at the RECODE refresher day(s)	1	0	0	0	0	1	0	0	1	0	0	1	0	1	1	1	1	8
Implementation/amending COPD protocol	0	0	0	0	0	0	0	0	0	0	1	0	1	1	1	1	1	6
More use of results from quality-of-life and COPD symptom questionnaires as part of consultation	0	1	0	0	1	1	0	0	1	1	1	1	1	0	1	1	1	11

*Self-management strategies*																		
More individual treatment plans are developed	0	0	0	0	0	0	1	0	1	1	1	1	1	1	1	0	1	9
Change in smoking cessation support	0	1	0	0	0	0	0	0	1	0	0	0	1	1	0	0	0	4
Early recognition of exacerbations	0	0	1	1	1	0	1	1	1	1	1	1	1	0	1	1	1	13
Change in motivational interviewing	0	1	0	0	0	0	1	0	0	0	0	0	1	0	0	0	0	3

*Clinical information system*																		
Initial use of the ICT support system Zorgdraad	0	0	0	0	0	1	1	0	1	1	0	1	0	1	0	1	1	8
Sustained use of the ICT support system Zorgdraad	0	0	0	0	0	0	0	0	0	0	0	0	0	0	0	0	0	0
Change in active identification and monitoring of high-risk COPD patients inside the practice, for example, using feedback reports	1	0	0	1	1	1	0	1	0	1	1	1	1	1	1	0	1	12

*Healthcare system*																		
Additional funding for physiotherapy	0	0	0	0	0	0	0	0	0	0	0	0	0	0	1	0	1	2
Total implementation score	2	4	4	4	6	6	7	7	8	9	9	10	11	12	12	13	14	
Starting level	3	1	3	1	3	1	2	2	2	3	1	1	2	1	1	1	1	

Abbreviations: COPD, chronic obstructive pulmonary disease; GP, general practitioner; ICT, information and communication technology; PCT, primary-care team.

**Table 3:** Level of integrated care experienced by the patients (PACIC) and healthcare provider (ACIC)

	*Baseline*		*12 Months*		*24 Months*		*Difference over 1 year*		*Difference over 2 years*	
	*Mean*	*s.d.*	*Mean*	*s.d.*	*Mean*	*s.d.*	*Mean*	*s.d.*	*Mean*	*s.d.*
*PACIC (*N*=436 (baseline), *N*=457 (12 months), *N*=353 (24 months))*										
Patient activation	2.31	1.26	2.29	1.14	2.33	1.16	−0.03	1.34	−0.08	1.34
Decision support	2.91	1.15	2.73	1.13	2.75	1.11	−0.19**	1.17	−0.25**	1.14
Goal setting	2.12	1.01	2.16	0.98	2.17	0.97	0.05	1.05	−0.05	1.03
Problem solving	2.22	1.15	2.26	1.13	2.27	1.15	0.04	1.18	−0.06	1.17
Follow-up	1.83	0.9	1.93	0.93	1.99	0.96	0.12*	0.98	0.08	0.96
Total PACIC score	2.28	0.95	2.26	0.94	2.31	0.96	−0.05	0.95	−0.05	0.95

*ACIC (*N*=20 (baseline), *N*=13 (12 months))*										
Organisation of healthcare system	5.49	2.57	6.63	1.67	—	—	1.32*	2.07	—	—
Community linkages	4.94	2.25	5.89	2.07	—	—	1.23*	1.53	—	—
Self-management	5.09	1.65	6.37	1.55	—	—	1.55*	1.87	—	—
Decision support	6.12	1.81	6.59	0.93	—	—	0.47	1.7	—	—
Delivery system design	6.24	1.96	6.32	1.54	—	—	0.32	2.06	—	—
Clinical information system	5.31	2.18	5.68	1.19	—	—	−0.04	1.98	—	—
Integration score	4.61	1.79	4.97	1.38	—	—	0.45	2.18	—	—
Total ACIC score	5.41	1.73	6.07	1.10	—	—	0.75	1.44	—	—

Abbreviations: ACIC, Assessment Chronic Illness Care; PACIC, Patient Assessment Chronic Illness Care.

*P<0.05 **P<0.01.

**Table 4:** The encountered barriers and facilitators of the multidisciplinary teams to their implementation of the RECODE programme

*Facilitators*	*Barriers*
*Individual factors*	
Improved knowledge of healthcare providers	Unmotivated patients for changing lifestyle because of underestimation of COPD symptoms
Motivated healthcare providers to change COPD care	Unmotivated healthcare providers for using ‘Zorgdraad’ because of unclear instructions, the inconvenient system and a lack of time to determine how ‘Zorgdraad’ works

*Social factors*	
The implementation experiences of the teams motivated and inspired other teams	Variability in adoption of ‘Zorgdraad’ between team members jeopardised the potential contribution of the ICT system to their purposes

*Organisational factors*	
Low starting level of integrated care results in room for improvements	Lack of adherence to the agreements between primary and secondary care
The practice-tailored feedback reports on patients’ health outcomes develop insight into own routines and patient needs	Small proportion of COPD patients who are in need of multidisciplinary treatment
	Staff turnover who followed the RECODE course(s)
	Problems with transferring information from ZORGDRAAD onto the different clinical information systems the practices used

*Broader societal factors*	
Better guidance and/or financial arrangements arranged by the care group to improve COPD care	Lack of reimbursement of exercise programmes and nutritional support
	Reimbursement of smoking cessation counselling and medication conditional on certain factors; when provided by healthcare providers who are registered as smoking cessation counsellors

Abbreviations: COPD, chronic obstructive pulmonary disease; ICT, information and communication technology.

**Table 5:** Multilevel models: influence of implementation on change in outcomes

	*Self-developed scale*^a^		*Δ PACIC^b^*		*Δ ACIC*^a^	
	β	N	β	N	β	N
Δ CCQ	0.001	327	−0.021	1629	0.004	297
Δ SGRQ	−0.138	308	−0.119	1624	0.492	284
Δ EQ-5D	0.004	330	−0.001	1701	−0.016	280
Δ MRC	0.074	345	−0.037	1733	−0.02	287
Δ MET minutes	94	310	173	1710	390	250
Δ SMAS, taking initiatives	0.01	309	1,211**	1719	1.004	251
Δ SMAS, investment behaviour	−0.228	310	1,349**	1712	0.781	252
Δ SMAS, self-efficacy	−0.013	308	0.592	1708	0.443	252

Abbreviations: ACIC, Assessment Chronic Illness Care; CCQ, Clinical COPD Questionnaire; EQ-5D, EuroQoL-5D; MET, metabolic equivalent time; MRC, Medical Research Council; PACIC, Patient Assessment Chronic Illness Care; SGRQ, St George’s Respiratory Questionnaire; SMAS, Self-Management Ability Scale.

*P<0.05, **P<0.01.

a Two-level models (patients nested in teams), correcting for starting score of different health outcomes and starting level of COPD care.

b Three-level models (measurement occasions nested in patients nested in teams), correcting for time, starting score of different health outcomes and level of COPD care.

**Table 6 tbl6:** 

*Intervention*	*Explanation of result*
*Delivery system design*	
Improved cooperation with physiotherapist(s)	The practice nurse, GP and physiotherapist(s) have agreed on the indications of referral, communication regarding patients and coordination of the treatment of COPD patients.
Improved cooperation with dietician(s)	The practice nurse, GP and dietician(s) have agreed on the indications of referral, communication regarding patients and coordination of the treatment of COPD patients.
Improved cooperation with lung specialist(s)	The practice nurse, GP and lung specialist(s) have agreed on the indications of referral, communication regarding patients and coordination of the treatment of COPD patients.
More multidisciplinary team meetings	Scheduled meetings regarding individual COPD patients, exchanging medical knowledge, and/or organisation of care with at least the GP, practice nurse and physiotherapists
Task re-allocation from GP to practice nurse or specialised nurse	The practice nurse has taken over tasks that were tasks of the GP before the start of the RECODE study.
Substitution of care from secondary to primary care	Primary healthcare providers have taken over tasks that were tasks of secondary healthcare providers before the start of the RECODE study.
Change in follow-up and visit structure	Patients visit the practice nurse or GP according to a structural follow-up plan.

*Decision support*	
Attendance of four disciplines at the initial RECODE course	Four different disciplines of healthcare providers (GP, practice nurse, physiotherapist, dietician) of the team attended the RECODE course.
Attendance of two or more disciplines at the RECODE refresher day(s)	Two or more healthcare providers from different disciplines attended the reunion.
Implementation / amending COPD protocol	The original COPD protocol is adapted or a new COPD protocol is developed and implemented.
More use of results from quality-of-life and COPD symptom questionnaires as part of consultation	The practice nurse started to use quality-of-life questionnaires (e.g., Clinical COPD Questionnaire (CCQ) or MRC) in consultation with patients

*Self-management strategies*	
More individual treatment plans are developed	Patients and practice nurses or GPs began to jointly formulate personal goals and these goals are recorded in the patient’s file.
Change in smoking cessation support	The practice nurse or GP pays different/more attention to smoking cessation than before the start of the RECODE study.
Early recognition of exacerbations	The practice nurse or GP pays more attention to teaching patients the early recognition of and the way to respond to exacerbations than before the start of the RECODE study.
Change in motivational interviewing	The practice nurse or GP started to use the motivational interviewing technique (more often) to understand and make use of patients’ personal goals in physical reactivation and lifestyle changes.

*Clinical information system*	
Initial use of the ICT support system Zorgdraad	The healthcare provider(s) actively tried to use Zorgdraad by logging into Zorgdraad and receiving individual instructions from an ICT implementation expert.
Sustained use of the ICT support system Zorgdraad	Using Zorgdraad after 12 months
Change in active identification and monitoring of high-risk COPD patients inside the practice, for example, using feedback reports	Active identification and monitoring of high-risk patients inside the practice (on the basis of the feedback reports).

*Healthcare system*	
Additional funding for physiotherapy	The practice used the supplementary funding provided by the local healthcare insurer for a COPD-specific exercise training programme for RECODE patients with MRC scores >2.

Abbreviations: CCQ, Clinical COPD Questionnaire; COPD, chronic obstructive pulmonary disease; GP, general practitioner; ICT, information and communication technology; MRC, Medical Research Council.

**Table 7 tbl7:** 

*Health outcomes*	N* (%) at baseline*	N *(%) at 12 months*	N* (%) at 24 months*
*Patient*			
PACIC	436 (79)	457 (82)	353 (64)
CCQ	553 (100)	515 (93)	394 (71)
SGRQ	550 (99)	496 (90)	372 (67)
EQ-5D	546 (99)	498 (90)	408 (74)
MRC	553 (100)	499 (90)	418 (75)
MET minutes	515 (93)	472 (85)	395 (71)
SMAS, taking initiatives	518 (94)	476 (86)	391 (71)
SMAS, investment behaviour	517 (93)	475 (86)	391 (71)
SMAS, self-efficacy	516 (93)	473 (85)	391 (71)

*Healthcare provider*			
12-month questionnaire	—	13 (65)	—
ACIC	20 (100)	13 (65)	—

Abbreviations: ACIC, Assessment Chronic Illness Care; CCQ, Clinical COPD Questionnaire; EQ-5D, EuroQoL-5D; MET, metabolic equivalent time; MRC, Medical Research Council; PACIC, Patient Assessment Chronic Illness Care; SGRQ, St George’s Respiratory Questionnaire; SMAS, Self-Management Ability Scale.

## Detailed description of the different interventions and results of the RECODE programme

[Table tbl6]

## Response rate

[Table tbl7]
